# Surgical and Functional Results of Hybrid 25-27-Gauge Vitrectomy Combined with Coaxial 2.2 mm Small Incision Cataract Surgery

**DOI:** 10.1155/2016/9186351

**Published:** 2016-02-04

**Authors:** Fabian Höhn, Florian Kretz, Mitrofanis Pavlidis

**Affiliations:** ^1^Helios Klinikum Pforzheim, Kanzlerstraße 2-6, 75175 Pforzheim, Germany; ^2^Augenklinik Ahaus, Am Schloßgraben 13, 48683 Ahaus, Germany; ^3^Augencentrum Köln, Josefstraße 14, 51143 Cologne, Germany

## Abstract

*Purpose*. To investigate outcomes after coaxial 2.2 mm small incision cataract surgery combined with hybrid 25-27-gauge vitrectomy in eyes with vitreoretinal disease and age-related cataract.* Methods*. A single-center, retrospective case series study of 55 subjects (55 eyes) with a mean age of 70 years who underwent combined small incision phacoemulsification, intraocular lens (IOL) implantation, and hybrid 25-27-gauge vitrectomy during the 12-month period to December 2014. Intraoperative and postoperative complications and visual results were the main outcome measures.* Results*. The mean follow-up period was 6 months (range: 2–18 months). Intraoperative findings were 3 retinal breaks (5.5%). No cases required corneal or scleral suture or conversion to larger-gauge vitrectomy. Postoperative complications consisted of posterior capsule opacification (12.7%), elevated intraocular pressure >30 mmHg (1.8%), and fibrin reaction (5.5%). There were no cases of hypotony (<7 mmHg), IOL decentration, or postoperative endophthalmitis. Visual acuity (mean ± SD) improved from 0.52 ± 0.6 logMAR preoperatively to 0.22 ± 0.46 logMAR at final postoperative visit (*P* < 0.0001).* Conclusion*. Surgical and visual outcomes suggest hybrid 25-27-gauge vitrectomy combined with small incision phacoemulsification and IOL implantation is feasible, safe, and effective as a one-step surgical procedure for the management of vitreoretinal pathologies and concurrent cataract.

## 1. Introduction

Small incision phacoemulsification and microincision cataract surgery (MICS) involving sub-2 mm clear corneal incisions are safe and effective standard surgical procedures [1–4]. The potential benefits of MICS relate to reduced wound leakage, good anterior chamber stability, and safety, minimizing surgically induced astigmatism, reducing higher-order corneal aberrations and promoting rapid postoperative wound healing [5, 6]. For treating vitreoretinal pathologies, transconjunctival sutureless microincision vitrectomy surgery (MIVS) using small-gauge (23-, 25-, or 27-gauge) instrumentation offers the potential for less inflammation, reduced operating time, and often faster visual rehabilitation after surgery compared with conventional 20-gauge vitrectomy [7–9]. Moreover, 25- and 27-gauge vitrectomy instrument system for MIVS effectively produces self-sealing sclerotomies that may alleviate concerns over wound sealing-related complications in selected vitreoretinal cases [10]. “Hybrid” is used to underline the mixed character of different seized infusion and working ports. Treating cataract and vitreoretinal pathologies in a combined one-step microincision phacovitrectomy procedure is an efficient well-tolerated technique that is becoming increasingly common [11–14]. Combined phacovitrectomy eliminates the need for a second operation, allows improved access to the retinal periphery during phacoemulsification, and offers potential for better vitrectomy outcomes [13, 15, 16].

The aim of this present interventional case series study was to retrospectively investigate and review surgical indications, intraoperative and postoperative complications, and visual acuity outcomes in eyes undergoing combined coaxial 2.2 mm small incision cataract surgery with intraocular lens (IOL) implantation and hybrid 25-27-gauge MIVS for the treatment of vitreoretinal disease and concurrent age-related cataract.

## 2. Materials and Methods

The authors report a single-center, retrospective, consecutive surgical case series that underwent small incision cataract surgery with IOL implantation combined with transconjunctival sutureless hybrid 25-27-gauge vitrectomy. All medical records and surgical charts of 102 patients (116 eyes) who underwent combined small-gauge phacovitrectomy surgery performed at Helios Klinikum Pforzheim, Pforzheim, Germany, between January and December 2014 were reviewed. Cases operated using 23-gauge vitrectomy or microincision coaxial phacoemulsification, where postoperative follow-up was less than 2 months, were excluded. Overall, 55 patients (55 eyes) were identified who had undergone coaxial small incision cataract surgery and IOL implantation combined with hybrid 25-27-gauge MIVS, who were all included in this study.

All patients in this series had preoperative lens opacification, which was graded mild or moderate in 36 of 55 eyes (65.5%). Demographic data and preoperative patient characteristics are presented in Table 1; surgical indication and cataract grade are shown in Table 2. Postoperative follow-up ranged between 2 months and 18 months (mean 6 months; standard deviation [SD] ±4.05). All patients were examined and assessed between 1 week and 4 weeks following the first postoperative day.

Combined phacovitrectomy procedures were carried out in single-session operations performed by the same surgeon, Fabian Höhn. Surgeries were completed throughout using a single phacovitrectomy console and the EVA ophthalmic surgical system (DORC International, Zuidland, Netherlands), together with a 25-gauge two-dimensional cutting (TDC) vitrectomy probe. The EVA surgical system is designed for use in anterior and posterior segment procedures that require infusion, vitreous cutting, aspiration, illumination, irrigation, lens emulsification and fragmentation, cautery, and diathermy as well as photocoagulation.

Preoperative data collected included patient demographics, visual acuity, intraocular pressure (IOP) measured in millimeters of mercury (mmHg) by Goldmann applanation tonometry, and diagnostic indication for combined phacovitrectomy surgery. Intraoperative data collected included suture placement if required, corneal incision and sclerotomy wound stability, and other complications observed during surgery. Postoperative visual acuity, IOP, degree of ocular inflammation, and IOL-related complications were analyzed.

### 2.1. Surgical Methods and Techniques

Following consultation and informed consent, patients underwent combined phacovitrectomy surgery under general anesthesia. Coaxial small incision cataract surgery was performed through a 2.2 mm corneal incision. A 27-gauge valved trocar (DORC) was preplaced in the inferior temporal quadrant 4 mm from the limbus, then a 2.2 mm clear corneal incision for cataract surgery was made at the 10-o'clock position, using a 2.2 mm ophthalmic phaco knife (MANI, Tochigi, Japan). For the side instrument, a 1.2 mm limbal incision was made at the 2-o'clock position left of the main incision using the same phaco knife. Following creation of clear corneal incision, viscoelastic material was injected into the anterior chamber.

**Figure 1:**
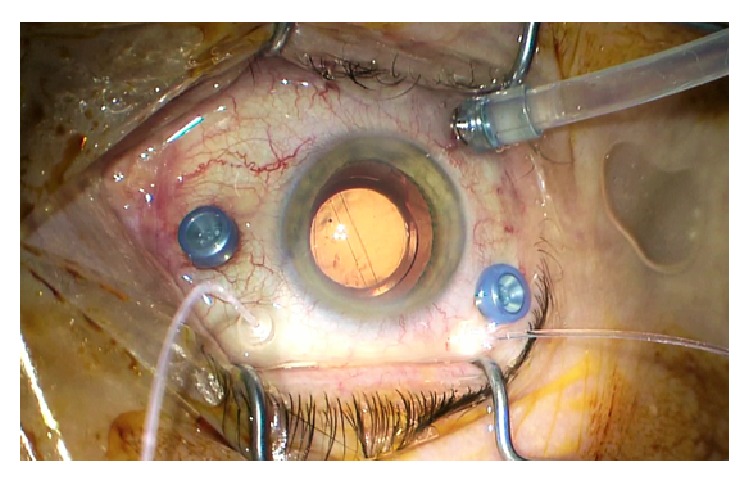
Hybrid 25-27-gauge vitrectomy setting following coaxial 2.2 mm small incision cataract surgery.

5 mm continuous curvilinear capsulorhexis was performed with microcapsulorhexis forceps suitable for 2.2 mm incision. After hydrodissection and rotation, a stop-and-chop phacoemulsification technique was utilized for nucleus removal. The cortex was removed and the capsular bag was filled with viscoelastic material. A hydrophilic, acrylic monofocal aspheric IOL, TECNIS iTec (Abbott Medical Optics AMO, Illinois, USA), was placed in the capsular bag by docking onto the inner lip of the main clear corneal incision. The corneal wound was hydrated with balanced salt solution following removal of viscoelastic material. The valve of the preplaced trocar was removed by surgical forceps, and the high-flow infusion line of the EVA surgical system was then connected. The eye was pressurized, allowing for controlled placement of two 25-gauge vitrectomy trocars in the superior quadrants 3.5 mm from the limbus. A 27-gauge twin light chandelier was placed at 11 and 1 o'clock position (Figure 1).

Vitreous surgery was performed using a 25-gauge TDC vitreous cutter controlled using the EVA vacuum vitrectomy unit. The vacuum level was placed at maximum 600 mmHg, and the vitreous cutter rate set at 8,000 cuts per minute (cpm). The TDC vitrectomy has a second port in the distal part of the inner pipe, which enables permanent aspiration, constant flow, and doubling of vitreous cut rate to an effective operating speed of 16,000 cpm. Vitrectomy was undertaken to achieve complete evacuation of the posterior vitreous and extensive removal of peripheral vitreous. In some patients, vitrectomy was combined with epiretinal membrane dissection and/or internal limiting membrane peeling. Fluid-air or gas exchange was performed to prevent postoperative hemorrhage and hypotonia in all cases.

Ocular surface preparation prior to surgery consisted of rinsing the conjunctival sac of the eye to be operated on with povidone-iodine and careful application of povidone-iodine via swabbing to the periocular skin of both eyes. No antibiotic agent was added to the irrigation fluid or anterior chamber. Patients received a course of topical ophthalmic corticosteroid therapy with dexamethasone together with gentamicin antibiotic treatment for between 4 weeks and 5 weeks postoperatively.

### 2.2. Data Analysis

A series of prespecified primary and secondary outcome measures were analyzed retrospectively. Primary intraoperative outcome measures were leaking corneal and scleral incision requiring suturing, posterior capsule tear, conversion to larger-gauge vitrectomy, and retinal break. Primary postoperative outcome measures were IOP change, fibrin in the anterior chamber, IOL capture or decentration, posterior capsule opacification (PCO), choroidal effusions, retinal or choroidal detachment, and endophthalmitis.

Visual acuity was assessed as the main secondary outcome measure. For statistical analysis, standard Snellen measurements were converted to logarithm of the minimum angle of resolution (logMAR) values. Preoperative and postoperative logMAR visual acuities were compared using the paired two-tailed Student's* t*-test method. In the case series analysis, a postoperative IOP reading of less than 7 mmHg was classified as hypotony.

## 3. Results

### 3.1. Study Population and Baseline Characteristics

A total of 55 patients (55 eyes) underwent combined small incision phacoemulsification, IOL implantation, and hybrid 25-27-gauge vitrectomy surgery. The average patient age was 70 years, with 23 male and 32 female subjects. The mean postoperative follow-up was 6 months (range: 2–18 months).

The most common indication for vitrectomy surgery was epiretinal membrane (26 eyes, 47.3%), followed by macular hole stage 4 (11 eyes, 20%), vitreomacular traction (6 eyes, 10.9%), and proliferative diabetic retinopathy (5 eyes, 9.1%). Internal tamponade was performed with 20% sulfur hexafluoride (SF6) or air; the decision and selection regarding tamponade procedure were based on assessment of preoperative and intraoperative clinical characteristics.

### 3.2. Primary Intraoperative and Postoperative Outcome Measures

Intraoperative and postoperative findings are shown in Table 3. None of the eyes in the case series required a corneal suture to seal the corneal tunnel, no sclerotomy sutures were needed, and all cases were completed without conversion to larger-gauge vitrectomy (23- or 20-gauge). A retinal break occurred in 3 eyes (5.5%). None of these 3 eyes had an iatrogenic retinal break, and the break was classified as a preexisting retinal break. All breaks were successfully managed with endolaser treatment using a curved 25-gauge endolaser probe (DORC).

The preoperative IOP (mean ± SD) was 15.2 ± 2.84 mmHg, and the postoperative IOP was 14.29 ± 6.96 mmHg. There were no cases of postoperative hypotony (IOP < 7 mmHg). One eye (1.8%) experienced elevated IOP greater than 30 mmHg on Day 1 after surgery, requiring topical hypotensive medications, and normalized IOP was achieved at the next examination.

Fibrin reaction in the anterior chamber was observed in 3 eyes (5.5%) the day after surgery, which was resolved following topical steroid treatment. During follow-up, there were no cases of lOL decentration or capture, while PCO developed in 7 eyes (12.7%). There were no cases of postoperative endophthalmitis or choroidal detachment.

### 3.3. Secondary Outcome Measures

The preoperative logMAR visual acuity (mean ± SD) in the current case series was 0.52 ± 0.6. At the final follow-up visit, logMAR visual acuity (mean ± SD) was 0.22 ± 0.46, which was a statistically significant improvement (*P* < 0.0001) from baseline, and represents an average improvement in visual acuity of 0.30 logMAR. Overall, at last postoperative follow-up visit, visual acuity had improved in 49 eyes (89.1%), was unchanged in 3 eyes (5.5%), and worsened in 3 eyes (5.5%). Monitored visual loss occurred as a result of progressive diabetic macular edema in one case and a conversion into exsudative age-related macular degeneration in two patients.

## 4. Discussion

In a series of 85 eyes, Canan et al. [17] found that phacovitrectomy using combined 20-gauge vitrectomy and 2.8 mm phacoemulsification with a standard phaco-chop technique was safe and effective for proliferative diabetic retinopathy. Developments in small incision cataract surgery together with enhancements in smaller-gauge vitrectomy instrumentation systems provide additional opportunities for securing effective and safe outcomes in combined phacovitrectomy for complex vitreoretinal diseases with simultaneous cataract [8, 14, 18–20]. Phacovitrectomy with either conventional 20-gauge vitrectomy or MIVS reduces surgical trauma for patients with vitreoretinal disease and cataract, while high-speed small-gauge vitrectomy cutters improve vitrectomy surgery by generating less vitreous traction and more efficient vitreous removal [21]. Moreover, studies confirm that phacovitrectomy improves visualization during the vitrectomy procedure in cases where there is a clinically significant lens opacity and speeds visual rehabilitation after surgery [22, 23].

The present clinical study was designed specifically to evaluate the potential intraoperative and postoperative complications and visual results of a hybrid 25-27-gauge microincisional sutureless vitrectomy in combination with coaxial small incision cataract surgery, and the primary and secondary outcomes have been reported above.

None of the 55 eyes in our case series required suture of the cornea wound or sclerotomy site at the end of the surgery, and there were no serious complications related to corneal wound leakage. A similar retrospective study which evaluated combined 1.8 mm microincision cataract surgery and 23-gauge vitrectomy found corneal suturing was required in 6 of 50 eyes (12%), with a sclerotomy suture in 4 eyes (8%) [15]. One possible explanation for the incidence of suturing could be the pressure force created during the insertion of a 23-gauge trocar instrument. For our case series, the 25- and 27-gauge trocars that were used require less insertion force than larger-sized trocars because they have a smaller diameter. Another contributing factor explaining sutureless procedures could be related to preplacement of the infusion trocar prior to creating a 2.2 mm tunnel incision. A ases series of 60 patients treated with combined 23-gauge phacovitrectomy found that vitrectomy ports were self-sealing in all eyes except 4 (6.7%) [24]. From another case series, Jalil et al. [16] reported that 4 of 43 cases (9.3%) required suturing of one or more ports during 23-gauge phacovitrectomy. The fact that in our series no eyes required scleral suturing suggests combined 25- and 27-gauge sclerotomies immediately self-seal following trocar removal, leading to faster visual rehabilitation and minimal ocular inflammation [24].

Intraoperative complications commonly associated with pars plana vitrectomy (PPV) procedures are iatrogenic retinal breaks, lens touch, and iatrogenic retinal tears [25]. There were 3 cases (5.5%) of intraoperative retinal break observed in our case series, although none of these eyes developed retinal detachment postoperatively. Higher incidences of retinal break during vitrectomy have been reported in the literature. Analysis of 2,471 primary PPV operations between 2001 and 2010 found that intraoperative iatrogenic retinal breaks developed in 10.09% of eyes overall, with an incidence of 32.45% in eyes with tractional retinal detachment and 16.3% of eyes with macular hole [26]. Risk factors include phakia and absence of a preoperative PVD [26, 27]. Intraoperative iatrogenic peripheral retinal breaks occurred in 15.2% (98 of 645 eyes) of cases involving 20-gauge PPV, approximately 4 in 10 breaks related to traction at sclerotomy entry site, in a large interventional case series study by Ramkissoon et al. [28]. Induction of PVD during vitrectomy is associated with a significantly higher incidence of retinal breaks [29, 30]. The frequency of retinal breaks related to the PPV operation was 6.9% in patients with epiretinal membrane and 14.6% in patients with macular hole, in a retrospective, comparative study by Chung et al. [29]. An intraoperative retinal break in 9 of 50 eyes (18%) undergoing MICS and 23-gauge vitrectomy for posterior segment disease was reported by Czajka et al. [15]. Prospective study data show that entry site retinal breaks are uncommon in patients undergoing small-gauge (23-, 25-gauge) vitrectomy, while a 2-year observational study involving a large series undergoing 20-gauge or 23-gauge vitrectomy found a significantly lower incidence of anterior iatrogenic retinal breaks in patients treated with the smaller-gauge surgery (7.8% versus 16.7% for 20-gauge vitrectomy) [31, 32].

No case of capsule tear was observed during phacovitrectomy surgery. Treumer et al. [33] reported posterior capsule tears in 7 of 111 eyes (6.3%) treated with combined PPV, phacoemulsification, and IOL implantation compared with 4 of 50 eyes (8%) in eyes that underwent sequential PPV and cataract surgery. An evaluation of 114 eyes undergoing combined 23-gauge phacovitrectomy between January 2006 and March 2009 found that capsular tears were more frequent in eyes with a prior history of radiation or vitrectomy [14]. Similar to the study presented here, a case series of 52 eyes that underwent combined MICS and PPV, mostly 23-gauge, reported posterior capsule rupture in 2 patients (3.8%) [16].

There were no occurrences of postoperative hypotony in the present study. In a smaller series of 30 eyes, Moon et al. [34] found a low risk of postoperative hypotony following combined 23-gauge sutureless vitrectomy and clear corneal phacoemulsification for rhegmatogenous retinal detachment repair. Only one eye (0.7%) experienced severe postoperative hypotony (<6 mmHg) despite the absence of suturing of sclerotomy sites, in an interventional cases series of 108 patients (136 eyes) with proliferative diabetic retinopathy who underwent combined 23-gauge phacovitrectomy [35]. A study evaluating 23-gauge phacovitrectomy using microincision phacoemulsification reported that hypotony (IOP < 9 mmHg) occurred in 18% (9/50) of eyes [15]. Oshima et al. [10] found that all sclerotomies were self-sealed without hypotony (IOP ≤ 7 mmHg) from Day 1 postoperatively in an experimental study evaluating a new 27-gauge instrument system for transconjunctival MIVS. No eyes in our series developed choroidal detachment postoperatively.

It was decided not to administer antibiotics to the anterior chamber at the end of the phacovitrectomy case; Delyfer et al. [36] reported that intracameral injection of high doses of cefuroxime at the end of uneventful cataract surgery induced anterior and posterior inflammation, with extensive macular edema associated with a large serous retinal detachment. There is nonetheless evidence of benefit that may justify the use of intracameral cefuroxime to reduce the rate of acute endophthalmitis after cataract surgery [37, 38].

Formation of posterior synechia of the iris is a postoperative complication of combined phacoemulsification and PPV. Oh et al. [39] identified postoperative synechia in 6.1% of 263 eyes treated with 23-gauge phacovitrectomy, which is a relatively low incidence when compared with other studies, with reported frequencies as high as 30% observed after phacovitrectomy in patients with proliferative diabetic retinopathy [40]. In our case series, 3 patients (5.5%) were identified with postoperative anterior chamber fibrin deposition, a known risk factor of posterior synechia, although no patient developed postoperative iris synechia in the present case series.

With regard to other postoperative anterior segment complications, the rate of posterior capsule opacification over the follow-up period was 12.7% (7 eyes), which is at the lower end of the range reported from similar investigations. Posterior capsule opacification is a common postoperative anterior segment complication associated with combined phacovitrectomy, with incidence rates of up to 51% reported in the literature [41]. Wensheng et al. [42] observed a PCO rate of 21.5% in 186 eyes of 149 patients who underwent combined phacoemulsification and vitrectomy for coexisting cataract and vitreoretinal diseases. Studies indicate a lower PCO rate in eyes undergoing transconjunctival 23-gauge phacovitrectomy compared with eyes treated using 20-gauge phacovitrectomy [43, 44]. Contributing factors for the development of PCO are increased surgical manipulation and inflammation, rhegmatogenous retinal detachment, gas tamponade, intraoperative/postoperative complications, and postoperative posturing [44]. Minimal fluid-air exchange during combined small-gauge phacovitrectomy may be beneficial in reducing the possibility of postoperative hypotony and IOL-related complications [45].

There were no cases of intraocular lens capture or decentration following combined phacovitrectomy surgery. A case series evaluation of sub-2 mm MICS combined with 23- or 20-gauge vitrectomy using an IOL with a 4-point fixation design similarly reported no cases of IOL decentration [16]. Compared with 25-gauge phacovitrectomy, more frequent IOL decentration has been observed with 20-gauge vitrectomy combined with phacofragmentation [46]. Better centration has been recorded with a 4-point haptic design IOL compared with an intraocular lens incorporating a 2-point haptic design [47]. In a comparative study, Leiderman et al. [48] revealed that single-piece acrylic IOLs are associated with a low rate of surgical complications after combined phacovitrectomy.

Visual results that were recorded in our study population are generally consistent with published outcomes from other clinical studies, demonstrating that good functional outcomes are achievable with combined hybrid MIVS and phacoemulsification using 2.2 mm microincision corneal wounds. Combining phacoemulsification, IOL implantation and vitrectomy offer clearer visualization during surgery compared with sequential procedures, and often time decreases visual rehabilitation time in cases with early or visually significant cataracts [22, 49]. Good success rates have been reported, with 95% of patients achieving a 2-line or greater improvement in visual acuity within 6 weeks of combined phacovitrectomy surgery in one institution in the United States [23].

To summarize, surgical and visual outcomes demonstrate that a single-session approach is safe, feasible, and effective for the treatment of vitreoretinal pathology and coexisting cataract, with minimal incremental surgical risk. Additional clinical studies evaluating multicenter practice outcomes utilizing combined phacovitrectomy will help guide practitioners as they transition toward more efficient minimally invasive combination approaches for a variety of vitreoretinal pathologies with and without visually significant cataract.

## Figures and Tables

**Table 1:** Demographic data and preoperative clinical features.

Variable	Data
Number of patients (eyes)	55 (55)
Gender (male : female)	23 : 32
Age (mean ± SD)	70.0 ± 10.33 years
Laterality (OD : OS)	26 : 29
Preoperative logMAR BCVA (mean ± SD)	0.52 ± 0.6

SD, standard deviation; OD, right eye; OS, left eye; logMAR, logarithm

of the minimum angle of resolution; BCVA, best-corrected visual acuity.

**Table 2:** Vitreoretinal indication and cataract grade.

Variable	Patients, *n* (%)
Diagnosis	
Rhegmatogenous retinal detachment	2 (3.6)
Epiretinal membrane	26 (47.3)
Macular hole stage 4	11 (20)
Vitreous hemorrhage	3 (5.5)
Vitreomacular traction	6 (10.9)
Proliferative diabetic retinopathy	5 (9.1)
Subretinal hemorrhage	2 (3.6)
Cataract grade	
Mild nuclear sclerosis ± cortical spoking	22 (40)
Moderate	14 (25.5)
Dense brunescent	12 (21.9)
Dense posterior subcapsular	3 (5.5)
Degree and type of cataract not recorded	4 (7.3)

**Table 3:** Main outcomes: intraoperative and postoperative findings.

Variable	Patients, *n* (%)
Intraoperative findings	
Retinal break	3 (5.5)
Posterior capsule tear	0 (0)
Corneal suture	0 (0)
Scleral suture	0 (0)
Conversion to larger-gauge vitrectomy	0 (0)
Postoperative findings	
Fibrin in the anterior chamber	3 (5.5)
Hypotony (<7 mmHg)	0 (0)
Elevated intraocular pressure (>30 mmHg)	1 (1.8)
Retinal or choroidal detachment	0 (0)
Endophthalmitis	0 (0)
Posterior capsule opacification	7 (12.7)
Intraocular lens capture or decentration	0 (0)

## References

[1] Kim E. C., Byun Y. S., Kim M. S. (2011). Microincision versus small-incision coaxial cataract surgery using different power modes for hard nuclear cataract. *Journal of Cataract and Refractive Surgery*.

[2] Alió J., Rodriguez-Prats J. L., Galal A. (2006). Advances in microincision cataract surgery intraocular lenses. *Current Opinion in Ophthalmology*.

[3] Dosso A. A., Cottet L., Burgener N. D., Di Nardo S. (2008). Outcomes of coaxial microincision cataract surgery versus conventional coaxial cataract surgery. *Journal of Cataract and Refractive Surgery*.

[4] Wilczynski M., Supady E., Loba P., Synder A., Omulecki W. (2011). Results of coaxial phacoemulsification through a 1.8-mm microincision in hard cataracts. *Ophthalmic Surgery Lasers and Imaging*.

[5] Chen C., Zhu M., Sun Y., Qu X., Xu X. (2014). Bimanual microincision versus standard coaxial small-incision cataract surgery: meta-analysis of randomized controlled trials. *European Journal of Ophthalmology*.

[6] Denoyer A., Denoyer L., Marotte D., Georget M., Pisella P.-J. (2008). Intraindividual comparative study of corneal and ocular wavefront aberrations after biaxial microincision versus coaxial small-incision cataract surgery. *British Journal of Ophthalmology*.

[7] Krishnan R., Tossounis C., Fung Yang Y. (2013). 20-Gauge and 23-gauge phacovitrectomy for idiopathic macular holes: comparison of complications and long-term outcomes. *Eye*.

[8] Eckardt C. (2005). Transconjunctival sutureless 23-gauge vitrectomy. *Retina*.

[9] Thompson J. T. (2011). Advantages and limitations of small gauge vitrectomy. *Survey of Ophthalmology*.

[10] Oshima Y., Wakabayashi T., Sato T., Ohji M., Tano Y. (2010). A 27-gauge instrument system for transconjunctival sutureless microincision vitrectomy surgery. *Ophthalmology*.

[11] Honjo M., Ogura Y. (1998). Surgical results of pars plana vitrectomy combined with phacoemulsification and intraocular lens implantation for complications of proliferative diabetic retinopathy. *Ophthalmic Surgery and Lasers*.

[12] Oshima Y., Ohji M., Tano Y. (2006). Surgical outcomes of 25-gauge transconjunctival vitrectomy combined with cataract surgery for vitreoretinal diseases. *Annals of the Academy of Medicine Singapore*.

[13] Schönfeld C.-L. (2013). 23- vs 20-gauge pars plana vitrectomy in combination with bimanual microincisional cataract surgery (b-MICS) for the treatment of macular hole and cataract as a one-step procedure. *Eye*.

[14] Sisk R. A., Murray T. G. (2010). Combined phacoemulsification and sutureless 23-gauge pars plana vitrectomy for complex vitreoretinal diseases. *British Journal of Ophthalmology*.

[15] Czajka M. P., Frajdenberg A., Johansson B. (2014). Outcomes after combined 1.8-MM microincision cataract surgery and 23-gauge transconjunctival vitrectomy for posterior segment disease: a retrospective study. *Retina*.

[16] Jalil A., Steeples L., Subramani S., Bindra M. S., Dhawahir-Scala F., Patton N. (2014). Microincision cataract surgery combined with vitrectomy: a case series. *Eye*.

[17] Canan H., Sizmaz S., Altan-Yaycioğlu R. (2013). Surgical results of combined pars plana vitrectomy and phacoemulsification for vitreous hemorrhage in PDR. *Clinical Ophthalmology*.

[18] Alió J., Rodríguez-Prats J. L., Galal A., Ramzy M. (2005). Outcomes of microincision cataract surgery versus coaxial phacoemulsification. *Ophthalmology*.

[19] Heath G., Rahman R. (2010). Combined 23-gauge, sutureless transconjunctival vitrectomy with phacoemulsification without face down posturing for the repair of idiopathic macular holes. *Eye*.

[20] Hütz W. W., Hoffmann P., Hengerer F. (2011). Fifty consecutive cases of transconjunctival sutureless 23-gauge vitrectomy combined with phacoemulsification and IOL implantation. *Ophthalmic Surgery Lasers and Imaging*.

[21] Teixeira A., Chong L. P., Matsuoka N. (2010). Vitreoretinal traction created by conventional cutters during vitrectomy. *Ophthalmology*.

[22] Theocharis I. P., Alexandridou A., Gili N. J., Tomic Z. (2005). Combined phacoemulsification and pars plana vitrectomy for macular hole treatment. *Acta Ophthalmologica Scandinavica*.

[23] Villegas V. M., Gold A. S., Latiff A. (2014). Phacovitrectomy. *Developments in Ophthalmology*.

[24] Sood V., Rahman R., Denniston A. K. (2009). Phacoemulsification and foldable intraocular lens implantation combined with 23-gauge transconjunctival sutureless vitrectomy. *Journal of Cataract and Refractive Surgery*.

[25] Jackson T. L., Donachie P. H. J., Sparrow J. M., Johnston R. L. (2013). Kingdom National Ophthalmology Database Study of Vitreoretinal Surgery: report 1; case mix, complications, and cataract. *Eye*.

[26] Dogramaci M., Lee E. J. K., Williamson T. H. (2012). The incidence and the risk factors for iatrogenic retinal breaks during pars plana vitrectomy. *Eye*.

[27] Tarantola R., Tsui J., Graff J. (2013). Intraoperative sclerotomy-related retinal breaks during 23-gauge pars plana vitrectomy. *Retina*.

[28] Ramkissoon Y. D., Aslam S. A., Shah S. P., Wong S. C., Sullivan P. M. (2010). Risk of iatrogenic peripheral retinal breaks in 20-G pars plana vitrectomy. *Ophthalmology*.

[29] Chung S. E., Kim K.-H., Kang S. W. (2009). Retinal breaks associated with the induction of posterior vitreous detachment. *American Journal of Ophthalmology*.

[30] Hikichi T., Kosaka S., Takami K. (2012). Incidence of retinal breaks in eyes undergoing 23-gauge or 20-gauge vitrectomy with induction of posterior vitreous detachment. *Retina*.

[31] Ehrlich R., Goh Y. W., Ahmad N., Polkinghorne P. (2012). Retinal breaks in small-gauge pars plana vitrectomy. *American Journal of Ophthalmology*.

[32] Jalil A., Ho W. O., Charles S., Dhawahir-Scala F., Patton N. (2013). Iatrogenic retinal breaks in 20-G versus 23-G pars plana vitrectomy. *Graefe's Archive for Clinical and Experimental Ophthalmology*.

[33] Treumer F., Bunse A., Rudolf M., Roider J. (2006). Pars plana vitrectomy, phacoemulsification and intraocular lens implantation. Comparison of clinical complications in a combined versus two-step surgical approach. *Graefe's Archive for Clinical and Experimental Ophthalmology*.

[34] Moon H., Sohn H. J., Lee D. Y. (2015). Combined 23-gauge sutureless vitrectomy and clear corneal phacoemulsification for rhegmatogenous retinal detachment repair. *International Journal of Ophthalmology*.

[35] Lee D. Y., Jeong H. S., Sohn H. J., Nam D. H. (2011). Combined 23-gauge sutureless vitrectomy and clear corneal phacoemulsification in patients with proliferative diabetic retinopathy. *Retina*.

[36] Delyfer M.-N., Rougier M.-B., Leoni S. (2011). Ocular toxicity after intracameral injection of very high doses of cefuroxime during cataract surgery. *Journal of Cataract and Refractive Surgery*.

[37] Sharma S., Sahu S. K., Dhillon V., Das S., Rath S. (2015). Reevaluating intracameral cefuroxime as a prophylaxis against endophthalmitis after cataract surgery in India. *Journal of Cataract and Refractive Surgery*.

[38] ESCRS Endophthalmitis Study Group (2007). Prophylaxis of postoperative endophthalmitis following cataract surgery: results of the ESCRS multicenter study and identification of risk factors. *Journal of Cataract and Refractive Surgery*.

[39] Oh J.-H., Na J., Kim S.-W., Oh J., Huh K. (2014). Risk factors for posterior synechiae of the iris after 23-gauge phacovitrectomy. *International Journal of Ophthalmology*.

[40] Shinoda K., O'hira A., Ishida S. (2001). Posterior synechia of the iris after combined pars plana vitrectomy, phacoemulsification, and intraocular lens implantation. *Japanese Journal of Ophthalmology*.

[41] Ling R., Simcock P., McCoombes J., Shaw S. (2003). Presbyopic phacovitrectomy. *British Journal of Ophthalmology*.

[42] Wensheng L., Wu R., Wang X., Xu M., Sun G., Sun C. (2009). Clinical complications of combined phacoemulsification and vitrectomy for eyes with coexisting cataract and vitreoretinal diseases. *European Journal of Ophthalmology*.

[43] Iwase T., Oveson B. C., Nishi Y. (2012). Posterior capsule opacification following 20- and 23-gauge phacovitrectomy (posterior capsule opacification following phacovitrectomy). *Eye*.

[44] Rahman R., Briffa B. V., Gupta A., Chinn D. J. (2011). Factors contributing to posterior capsule opacification following 23-gauge transconjunctival phacovitrectomy. *Ophthalmic Surgery Lasers and Imaging*.

[45] Nam D. H., Ku M., Sohn H. J., Lee D. Y. (2010). Minimal fluid-air exchange in combined 23-gauge sutureless vitrectomy, phacoemulsification, and intraocular lens implantation. *Retina*.

[46] Romero-Aroca P., Almena-Garcia M., Baget-Bernaldiz M., Fernández-Ballart J., Méndez-Marin I., Bautista-Perez A. (2009). Differences between the combination of the 25-gauge vitrectomy with phacoemulsification versus 20-gauge vitrectomy and phacofragmentation. *Clinical Ophthalmology*.

[47] Mingels A., Koch J., Lommatzsch A., Pauleikhoff D., Heiligenhaus A. (2007). Comparison of two acrylic intraocular lenses with different haptic designs in patients with combined phacoemulsification and pars plana vitrectomy. *Eye*.

[48] Leiderman Y. I., Andreoli M. T., Sun B., Dawood S. (2015). Pars plana vitrectomy combined with cataract extraction: a comparison of surgical outcomes using single-piece and multipiece foldable intraocular lenses. *Retina*.

[49] Muselier A., Dugas B., Burelle X. (2010). Macular hole surgery and cataract extraction: combined vs consecutive surgery. *American Journal of Ophthalmology*.

